# The transverse diameter of right common femoral vein by ultrasound in the supine position for predicting post-spinal hypotension during cesarean delivery

**DOI:** 10.1186/s12871-021-01242-8

**Published:** 2021-01-20

**Authors:** Shi-Fa Yao, Yan-Hong Zhao, Jing Zheng, Jie-Yan Qian, Chen Zhang, Zifeng Xu, Tao Xu

**Affiliations:** 1 Department of B ultrasound, the International Peace Maternity and Child Health Hospital, School of Medicine, Shanghai Jiao Tong University, Shanghai, China; 2 Shanghai Key Laboratory of Embryo Original Diseases, Shanghai, China; 3 Department of Anesthesiology, the International Peace Maternity and Child Health Hospital, School of Medicine, Shanghai Jiao Tong University, Shanghai, China; 4 Department of Obstetrical Ward, the International Peace Maternity and Child Health Hospital, School of Medicine, Shanghai Jiao Tong University, Shanghai, China; 5 Department of Biostatistics, the International Peace Maternity and Child Health Hospital, School of Medicine, Shanghai Jiao Tong University, Shanghai, China

**Keywords:** Transverse diameter, Right common femoral vein, Hypotension, Cesarean delivery, Ultrasound

## Abstract

**Background:**

Post-spinal anesthesia hypotension during cesarean delivery is caused by decreased systemic vascular resistance due to the blockage of the autonomic nerves, which is further worsened by inferior vena cava (IVC) compression by the gravid uterus. This study aimed to assess whether peak velocity and diameter of the IVC below the xiphoid or right common femoral vein (RCFV) in the inguinal region, as measured on ultrasound, could reflect the degree of IVC compression and further identify parturients at risk of post-spinal hypotension.

**Methods:**

Fifty-six parturients who underwent elective cesarean section with spinal anesthesia were included in this study; peak velocities and anteroposterior diameters of the IVC and peak velocities and transverse diameters of the RCFV were measured using ultrasound before anesthesia. The primary outcome was the ultrasound measurements of IVC and RCFV acquired before spinal anesthesia and their association with post-spinal hypotension. Hypotension was defined as a drop in systolic arterial pressure by > 20% from the baseline. Multinomial logistic regression analysis was used to identify the association between the measurements of IVC, RCFV, and post-spinal hypotension during cesarean delivery. Receiver operating characteristic curves were used to test the abilities of the identified parameters to predict post-spinal hypotension; the areas under the curve and optimum cut-off values for the predictive parameters were calculated.

**Results:**

A longer transverse diameter of the RCFV was associated with the occurrence of post-spinal hypotension (odds ratio = 2.022, 95% confidence interval [CI] 1.261–3.243). The area under the receiver operating characteristics curve for the prediction of post-spinal hypotension was 0.759 (95% CI 0.628–0.890, *P* = 0.001). A transverse diameter of > 12.2 mm of the RCFV could predict post-spinal hypotension during cesarean delivery.

**Conclusions:**

A longer transverse diameter of RCFV was associated with hypotension and could predict parturients at a major risk of hypotension before anesthesia.

**Trial registration:**

This study was registered at http://www.chictr.org.cn on 16, May, 2018. No. ChiCTR1800016163.

## Background

The incidence of post-spinal anesthesia hypotension during cesarean delivery is approximately 70% [1, 2]. Post-spinal anesthesia hypotension can lead to adverse maternal and fetal outcomes, such as maternal nausea, vomiting, dyspnea, neonatal depressed Apgar scores, and fetal acidosis [3–8]. Therefore, effective prediction of maternal hypotension could have immense clinical importance.

Post-spinal anesthesia hypotension during cesarean delivery is caused by decreased systemic vascular resistance due to the blockage of the autonomic nerves; the compression of the inferior vena cava (IVC) by the gravid uterus further worsens this hypotension. The compression of the IVC leads to reduced venous return, which in turn decreases the IVC diameter [9, 10].

The right common femoral vein (RCFV), the main extension of the right external iliac vein, is the sub-branch of the IVC. As the RCFV is close to the body surface, it can be easily detected by a high-frequency probe. More importantly, the RCFV is located at the distal part of the aortocaval compressed point; therefore, we hypothesized that the peak velocity and diameter of the RCFV would be more significant than the indirect parameters of the IVC below the xiphoid in women at high risk of hypotension during cesarean delivery, following spinal anesthesia.

The study aimed to assess whether peak velocity and anteroposterior diameter of the IVC below the xiphoid, or the peak velocity and transverse diameter of the RCFV in the inguinal region could reflect the degree of aortocaval compression, and further identify parturients at a major risk of post-spinal hypotension during elective cesarean delivery.

## Methods

### Materials and methods

After obtaining approval from the Research Ethics Committee of the International Peace Maternity and Child Health Hospital (Ethical number: GKLW 2017–85) and registering this prospective, observational study at http://www.chictr.org.cn (ChiCTR1800016163). A total of 58 parturients, aged 18–40 years, with a full-term (> 37-week gestation) singleton pregnancy, a height of 156–170 cm, and an American Society of Anesthesiology (ASA) score of I–II who underwent elective cesarean delivery with combined spinal epidural (CSE) anesthesia between January 2019 and June 2019, were recruited. All study participants provided signed informed consent. The exclusion criteria were as follows: an ASA score of III–IV, contraindications to spinal anesthesia, prolonged pregnancy (> 42 weeks), preexisting or pregnancy-induced hypertension or preeclampsia, placenta previa, placental abruption, multiple pregnancy, morbid obesity (body mass index [BMI] ≥36), fetal distress or fetal abnormalities, emergent cesarean delivery, and parturient refusal.

**Fig. 1:**
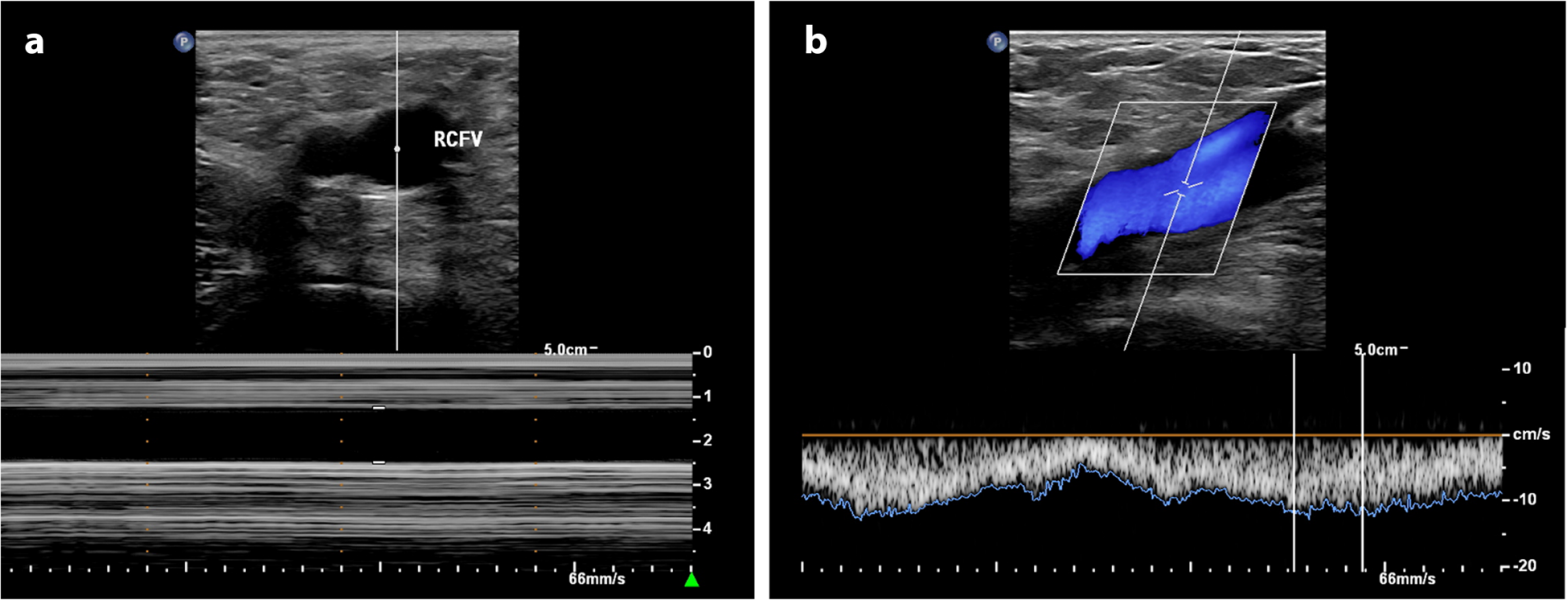
Measurement of RCFV by ultrasound with the high frequency linear array probe. **a** M-mode image showing the transverse diameter of RCFV; **b** Pulsed-wave Doppler-mode image showing the peak velocity of RCFV. RCFV, right common femoral vein

**Fig. 2:**
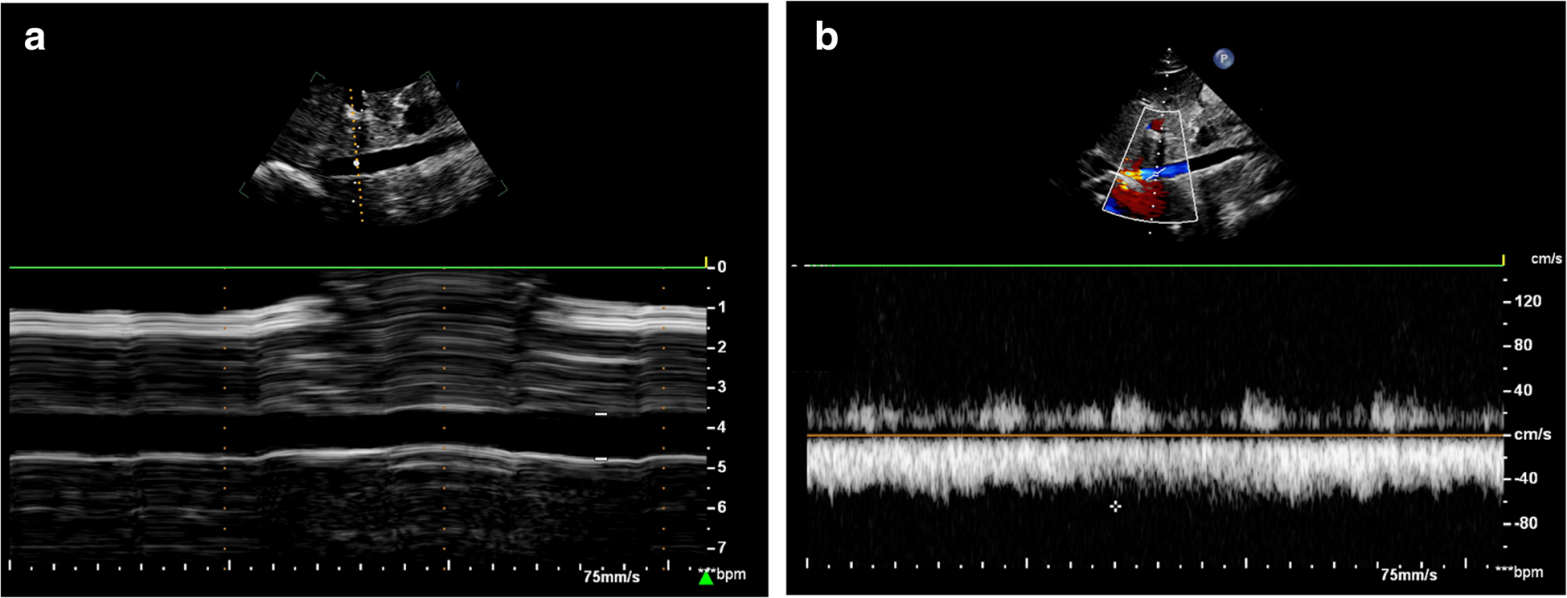
Measurement of IVC by ultrasound with the cardiac probe. **a** M-mode image showing the anteroposterior diameter of IVC; **b** Pulsed-wave Doppler-mode image showing the peak velocity of IVC; IVC, inferior vena cava

Parturients were instructed to fast for at least 6 h before the cesarean surgery. The ultrasound measurements were performed with the parturient on the transfer bed in the post-anesthesia care unit 15 min before anesthesia. An ultrasound device (EPIQ7; Philips, Ultrasound, Bothell, WA, USA) with a high-frequency linear array probe (L12–5; 5–12 MHz) was used for the measurement of the transverse diameter and peak velocity of the RCFV (Fig. 1). A cardiac probe (SC-1; 5–1 MHz) was used to measure the anteroposterior diameter and peak velocity of the IVC (Fig. 1). Parturients were in a supine position when the ultrasound examination was performed. Measurement sequences for the transverse diameter of the RCFV, peak velocity of the RCFV, anteroposterior diameter of the IVC, peak velocity of the IVC were always applied to ensure that all examinations were completed within 15 min. The transverse diameters and peak velocities of the RCFV were measured 1 cm proximal to the confluence of the great saphenous vein into the common femoral vein during end expiration (Fig. 1). The cardiac probe was placed below the xiphoid. The anteroposterior diameters and peak velocities of the IVC were measured 2–3 cm below the IVC-right atrial junction during end expiration (Fig. 2). Transverse and anteroposterior diameters were measured by M-mode ultrasound, and the M-mode sample line was always adjusted to pass through the center of vessels to measure the diameters more precisely (Fig. 1a and Fig. 2a). All peak velocities were measured using the pulsed-wave Doppler ultrasound mode. The Doppler sampling volume was placed in the center of the blood vessel, and the width of the sampling range gate was 2 mm. Doppler angle correction was performed when measuring velocity, with the calibration main line parallel to the direction of blood flow and at an angle of 50–60° (Fig. 1b and Fig. 2b). All the ultrasound recordings were performed by a board-certified ultrasound specialist; the anesthesiologists and parturients were blinded to the examination results.

The parturient was then transferred to the operating room. After entering the room, an intravenous (IV) line was established with an 18-G IV catheter in the dorsum or wrist vein of the right hand. Standard monitoring with electrocardiography, non-invasive blood pressure, and pulse oximetry were performed continuously. The cuff of the automated non-invasive blood pressure monitor was attached to the left arm. Systolic arterial pressure (SAP), heart rate (HR), and pulse oximetry were measured once per minute. The first two resting SAP and HR measurements with the parturient in the supine position were recorded, and the average values were recorded as baseline SAP and HR measurements. If the baseline SAP was above 140 mmHg, the parturient was excluded from the study because of suspected hypertension. A CSE puncture was performed at the L3–4 level with the parturient in the right lateral decubitus position. After the cerebrospinal fluid was detected, 0.75% isobaric ropivacaine (12 mg) with fentanyl (10 μg) was injected intrathecally via a 27-G Whitacre needle and an epidural catheter was inserted via an 18-G Tuohy needle by advancing it 3 cm into the epidural space. The parturient was moved immediately to a supine position with left uterine displacement by placing a wedge under the right hip before delivery. Moreover, lactated Ringer’s solution, at an open, co-loaded infusion rate of 1 mL/kg/min, was administered until delivery. Parturients were excluded from the study if they could still feel the pinprick sensation below the T6 level at the beginning of surgery. Epidural boluses of 5 mL lidocaine were administered intermittently until satisfactory anesthesia was achieved.

Hypotension was defined as a drop in SAP by > 20% from the baseline value before delivery. If hypotension occurred, a rescue phenylephrine bolus of 50 μg was administered by the anesthesiologist, and the phenylephrine bolus was administered every time the parturient presented with hypotension before delivery. Bradycardia was defined as a HR below 50 beats per minute (bpm). If bradycardia was identified, 0.5 mg of atropine was administered. After delivery, the Apgar scores at 1 and 5 min as well as the neonatal body weight were recorded. Further, 1 mL of umbilical artery (UA) blood was collected by the obstetrician immediately after delivery, and blood gas assessments were performed using a blood gas analyzer (iSTAT1 Analyzer MN:300-G; Abbott Point of Care Inc., Princeton, NJ, USA) with an iSTAT CG4 + test cartridge.

The primary outcome was the peak velocity and anteroposterior diameter values of the IVC below the xiphoid, the peak velocity and transverse diameter values of the RCFV as measured by ultrasound before anesthesia and the association between these measurements and post-spinal hypotension during cesarean delivery. Patient and obstetric characteristics, such as age, weight, height, BMI, gravidity, parity, gestational weeks, induction-delivery interval, upper sensory level, total IV fluid administered before delivery, total dose of phenylephrine and atropine, neonatal body weight, 1 and 5 min Apgar scores, and the pH of UA blood, were also recorded.

### Statistical analysis and sample size calculation

Based on our previous study, the odds ratio (OR) of the association between the perfusion index (PI) on the right toe and post-spinal hypotension during cesarean delivery was 0.49 (95% confidence interval [CI] 0.32–0.75, *P* = 0.0001) [2]. A logistic regression OR of 2–2.5 (equal to an OR of 0.4–0.5) was assumed in this study. To measure the OR at a power of 0.9, a two-tailed α of 0.05, and with a baseline prevalence of 40%, it was estimated that this study needed a minimal sample size of 52 parturients [11]. Considering a dropout rate of 10%, a sample size of 58 was required.

The patient and obstetric characteristics are presented as mean ± standard deviation or median (interquartile range), as appropriate, and were analyzed by an unpaired Student’s t-test, Fisher’s exact probability test, or Pearson’s Chi-Square test, as appropriate.

The parameters of the IVC and RCFV, measured by ultrasound in the supine position, were analyzed by multinomial logistic regression analysis to determine if they were independently associated with the incidence of post-spinal hypotension. Thereafter, the area under the receiver operating characteristic (ROC) curves was used to test the ability of the identified parameters to predict post-spinal hypotension, and the area under the curve (AUC) was calculated. The AUC is a measure of the accuracy of a parameter (AUC ≤ 0.5 indicates no predictive ability and AUC = 1.0 indicates the best possible prediction). The maximal value of Youden’s index was used as the criterion for selecting the optimum cut-off values of the predictive parameters; Youden’s index was calculated as follows:

Youden’s index = sensitivity + specificity – 1.

The perioperative hemodynamic parameters were assessed by two-way analysis of variance with the Bonferroni post hoc test.

All statistical analyses were performed using SPSS for Windows, version 24.0 (SPSS Inc., Chicago, IL, USA). Statistical significance was set at *P* < 0.05.

## Results

A total of 122 parturients were included in this study, and 64 parturients were excluded because they did not meet the inclusion criteria, they declined to participate, or because an ultrasound operator was not available. Ultrasound measurements were completed successfully in the remaining 58 parturients. Two parturients showed a sensory level below T6 at the beginning of the surgery. Finally, 56 parturients were followed up and analyzed.

Hypotension occurred in 24 (43%) parturients. Patient characteristics in the hypotension and no-hypotension groups are presented in Table 1. Maternal BMI and neonatal body weight were higher in the hypotension group (*P* = 0.04 and 0.015, respectively) than in the No-hypotension group. There was no significant difference with respect to age, weight, height, gravity, parity, gestational age, baseline SAP, and HR (Table 1).

**Table 1:** Patient Characteristics

	Hypotension (*n* = 24)	No hypotension (*n* = 32)	*P* value
Age (year)	32.5 ± 4.5	32.6 ± 4.1	0.986
Weight (kg)	74.2 ± 9.7	69.5 ± 7.3	0.050
Height (cm)	163.2 ± 3.9	163.4 ± 4.7	0.860
BMI	27.9 ± 3.5	26.1 ± 2.8	0.040
Gravity (n)	^a^2(1–2.75)	^a^1(1–2)	0.155
Parity (n)	^a^0(0–0)	^a^0(0–0)	0.571
Gestational age (weeks)	39.1 ± 1.1	38.7 ± 0.8	0.120
Baseline SAP (mmHg)	123 ± 12	121 ± 9	0.530
Baseline HR (bpm)	84 ± 12	83 ± 11	0.891
Neonatal body weight (g)	3712 ± 415	3416 ± 464	0.015

Values are mean ± SD or ^a^median (IQR). *BMI* Body mass index, *SAP* Systolic arterial pressure, *bmp* beats per minute, *SD* Standard deviations, *IQR* Interquartile range

Table 2 shows the anteroposterior diameter and peak velocity of the IVC, and the transverse diameter and peak velocity of the RCFV, in the Hypotension and No-hypotension groups. The transverse diameter of the RCFV in the Hypotension group was significantly longer than that in the No-hypotension group (*P* = 0.000).

**Table 2:** The parameters of vessels probed by ultrasound between two groups

	Hypotension (*n* = 24)	No hypotension (*n* = 32)	*P* value
AP diameter of IVC (mm)	12.0 ± 2.2	11.5 ± 1.6	0.227
Peak velocity of IVC (cm/s)	33.6 ± 15.7	39.1 ± 16.3	0.094
Transverse diameter of RCFV (mm)	12.8 ± 1.7	11.2 ± 1.4	0.000
Peak velocity of RCFV (cm/s)	8.2 ± 3.5	8.5 ± 4.9	0.810

Values are mean ± SD. *AP* Anteroposterior, *IVC* Inferior vena cava, *RCFV* Right common femoral vein, *SD* Standard deviations

Table 3 shows that the transverse diameter of the RCFV, as measured by ultrasound with the parturient in the supine position, was associated with the occurrence of post-spinal hypotension during cesarean delivery (OR = 2.022, 95% CI 1.261–3.243, *P* = 0.003). Other parameters measured by ultrasound were not associated with the occurrence of post-spinal hypotension.

**Table 3:** Results of multinomial logistic regression analysis to predict the incidence of post-spinal hypotension during elective cesarean delivery

	OR	95% CI		*P* value
		Lower limit	Upper limit	
AP diameter of IVC	0.824	0.642	1.056	0.125
Peak velocity of IVC	1.029	0.997	1.062	0.079
Transverse diameter of RCFV	2.022	1.261	3.243	0.003
Peak velocity of RCFV	1.063	0.903	1.250	0.465

*AP* Anteroposterior, *IVC* Inferior vena cava, *RCFV* Right common femoral vein, *OR* odds ratio, *95% CI* 95% confidential interval

**Fig. 3:**
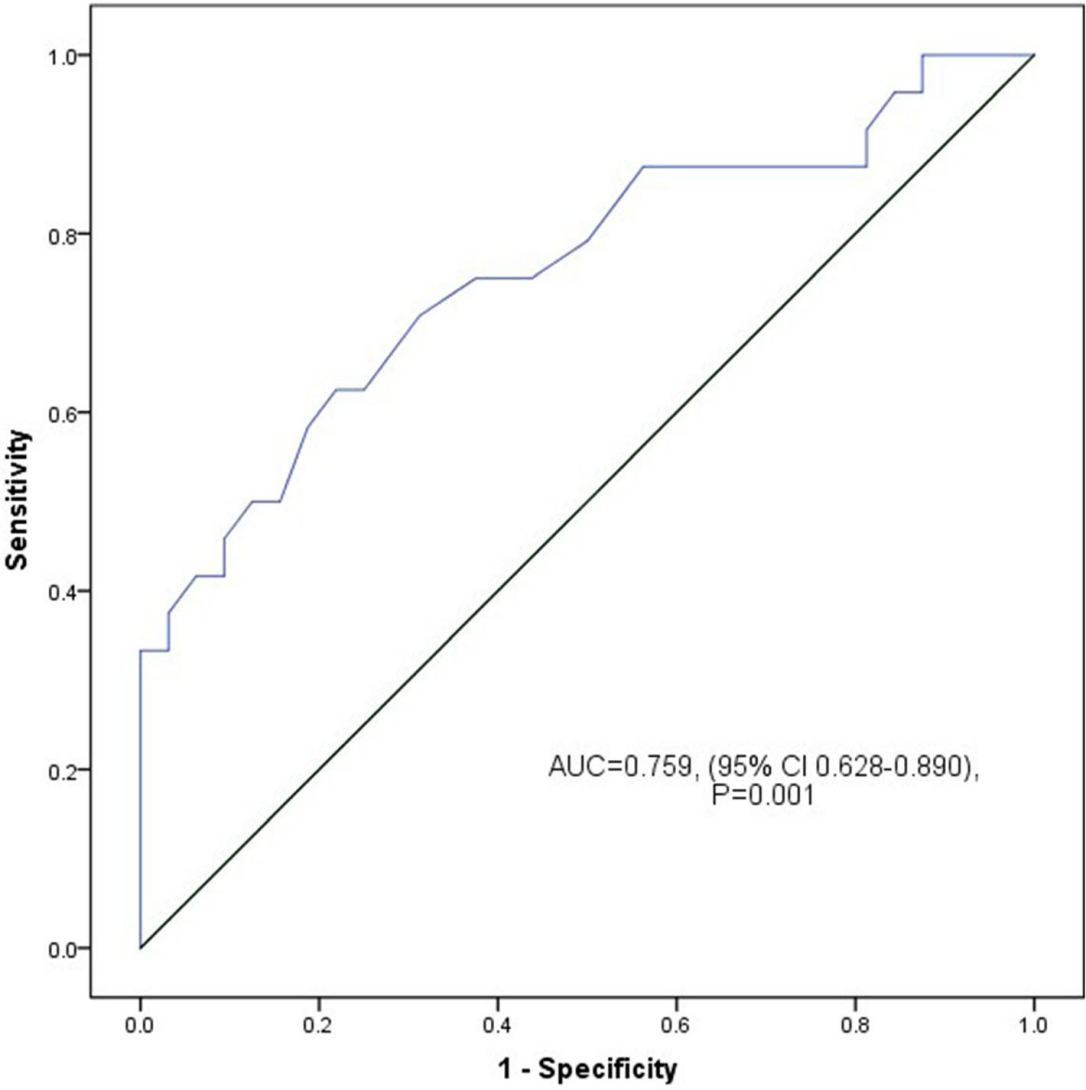
Receiver operating characteristics (ROC) curve of transverse diameter of RCFV before the spinal anesthesia for cesarean delivery. The optimal cut-off value for for predicting the incidence of hypotension in RCFV was 12.2 mm, area under the ROC curve, with 95% CIs showed in the figure. RCFV, right common femoral vein

The ROC analysis revealed that the transverse diameter of the RCFV with the parturient in the supine position was suitable for the prediction of parturients at risk of hypotension (AUC = 0.759, 95% CI 0.628–0.890, *P* = 0.001) (Fig. 3). The optimum cut-off point was 12.2 mm on maximum Youden index (sensitivity, 62.5%; specificity, 78.1%; positive-predictive value, 68.2%; and negative-predictive value, 73.5%).

Table 4 shows the obstetric characteristics grouped by the diameter of RCFV. The total dose of phenylephrine administered was significantly higher in the group with RCFV > 12.2 mm than that in the group with RCFV diameter ≤ 12.2 mm (*P* = 0.004). There was no significant difference in the induction-to-delivery interval, upper sensory level, total fluid before delivery, total dose of atropine administered, surgery time, neonatal body weight, 1 and 5 min Apgar scores, and pH of UA blood between the two groups.

**Table 4:** Obstetric characteristics by the diameter of RCFV

	RCFV> 12.2 mm (*n* = 22)	RCFV≤12.2 mm (*n* = 34)	*P* value
Induction-delivery interval (min)	11.5 ± 3.0	11.6 ± 3.7	0.889
Upper sensory level	^a^T5 (T4-T6)	^a^T6 (T4-T6)	0.495
Total intravenous fluid before delivery (mL)	366 ± 86	360 ± 88	0.814
Total dose of phenylephrine (μg)	^a^50(0–100)	^a^0(0–50)	0.004
Total dose of atropine (mg)	^a^0(0–0)	^a^0(0–0)	1.000
Surgery time (min)	46.6 ± 15.1	43.6 ± 15.9	0.487
Neonatal body weight (g)	3563 ± 437	3530 ± 486	0.799
1 min Apgar	^a^10 (10–10)	^a^10 (10–10)	0.555
5 min Apgar	^a^10 (10–10)	^a^10 (10–10)	1.000
Umbilical artery pH	7.31 ± 0.02	7.32 ± 0.03	0.282

Values are mean ± SD or ^a^median (IQR). *min* minute, *SD* Standard deviations, *IQR* Interquartile range

**Fig. 4:**
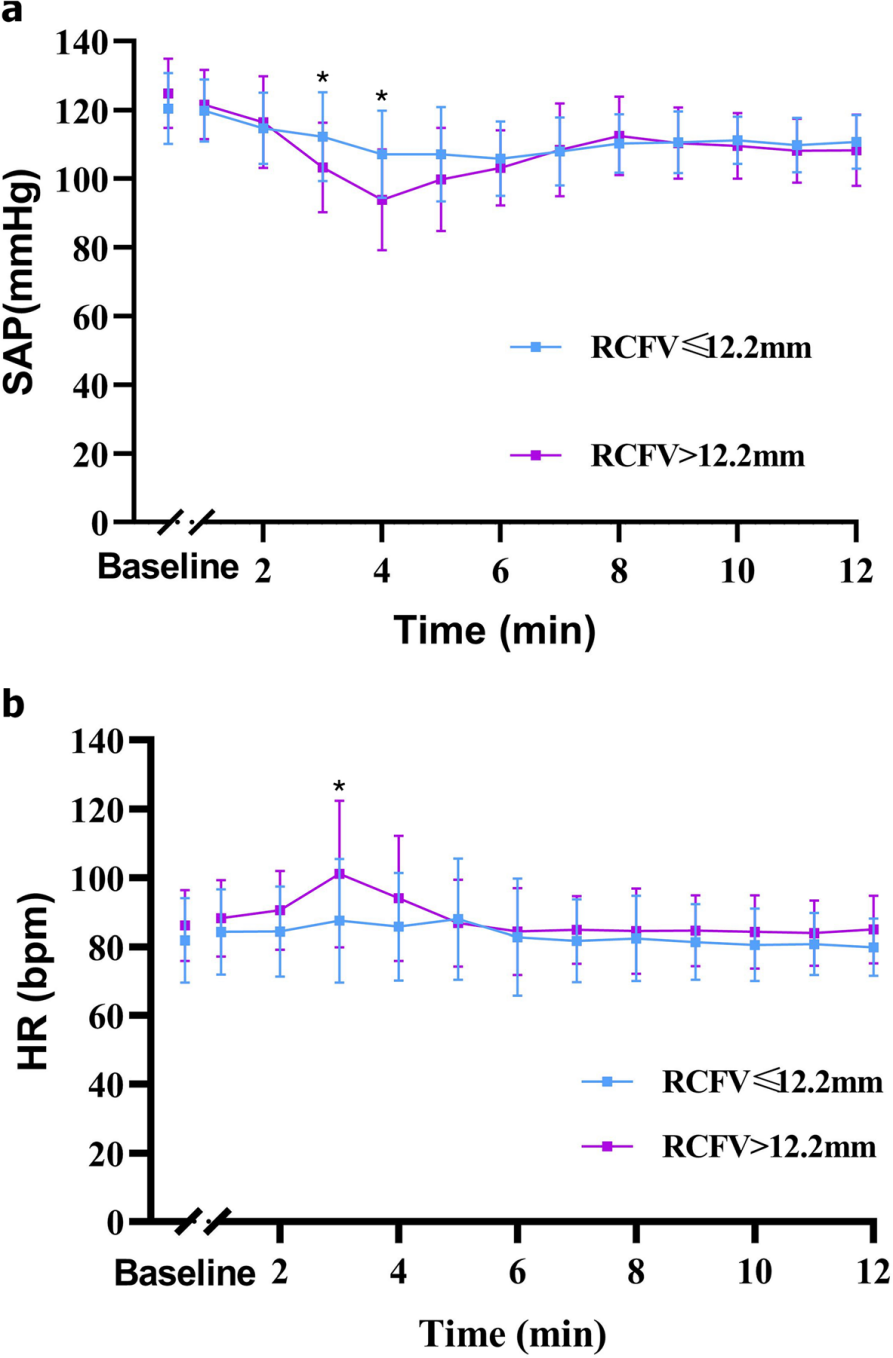
Serial changes of the SAP and HR over time after the spinal anesthesia. *Statistically significant difference between the two groups were assessed by two-way analysis of variance with the Bonferroni post hoc test. SAP: systolic arterial pressure; HR, heart rate; bpm, beats per minute

Although SAP decreased in both the groups (RCFV diameter > 12.2 mm and RCFV diameter ≤ 12.2 mm), SAP decreased markedly in the group with RCFV diameter > 12.2 mm in the time point of 3 and 4 min after spinal anesthesia (*P* = 0.015 and 0.001, respectively; Fig. 4a). Moreover, HR increased significantly in the group with RCFV diameter > 12.2 mm in the time point of 3 min after spinal anesthesia (*P* = 0.013; Fig. 4b).

## Discussion

In this study, the anteroposterior diameter and peak velocity of the IVC, as well as the transverse diameter and peak velocity of the RCFV, were measured by ultrasound before the spinal anesthesia. We hypothesized that the peak velocity and diameter of the RCFV would have more significance than the indirect parameters of the IVC below the xiphoid in women at a high risk of hypotension during cesarean delivery, following spinal anesthesia. Therefore, the transverse diameter of the RCFV was associated with the occurrence of hypotension after spinal anesthesia during cesarean delivery, and a cut-off value of > 12.2 mm could be used to predict subsequent hypotension.

Many studies have demonstrated that the velocity or diameter of the compressed IVC could indicate the degree of aortocaval compression by the gravid uterus, and could change dramatically in the supine position during late pregnancy using magnetic resonance imaging (MRI) [12–15]. Lee et al. also demonstrated a significant decrease in cardiac output on suprasternal Doppler, in late pregnancy, with parturients in the supine position, owing to IVC compression by the uterus [16]. However, none of these studies further clarified the relationship between the degree of IVC compression and the incidence of hypotension after spinal anesthesia during cesarean delivery. The anteroposterior diameters and velocities of the IVC below the xiphoid at the proximal end of the compressed IVC, measured by cardiac probe, were chosen as the indirect parameters of the degree of compression of the IVC in this study; this was because the compressed IVC and its main branches were under the gravid uterus or located in the pelvic cavity, which made them difficult to probe on ultrasound. However, these indirect parameters of the IVC could not reflect the real compression degree of the IVC by the uterus. Thus, none of the IVC parameters could be used as predictors for hypotension after spinal anesthesia during cesarean delivery.

The RCFV, the main extensions of the right external iliac vein, is the sub-branch of the IVC. As the RCFV is close to the body surface, it can be easily detected by a high-frequency probe. More importantly, the RCFV is located at the distal part of the aortocaval compression point; therefore, the peak velocity and diameter of the RCFV would theoretically decrease and increase, respectively, when the IVC was compressed. Therefore, we hypothesized that the parameters of the RCFV, measured by ultrasound, would have more significance than the indirect parameters of the IVC below the xiphoid in women at high risk of hypotension during cesarean delivery, following spinal anesthesia. Finally, the result of this study further improved our hypothesis.

Many studies have suggested that pre-operative baseline vascular tone and central blood volume are related to the incidence of hypotension during cesarean delivery, following spinal anesthesia. Thus, the PI, pleth variability index (PVI), HR, or heart rate variability (HRV) were used to predict hypotension. Both Toyama [9] and Duggappa [17] demonstrated that a baseline PI of the index finger of > 3.5 could predict the incidence of spinal anesthesia-induced hypotension during cesarean delivery. Sun [18] found that a greater baseline PVI was associated with hypotension after spinal anesthesia for cesarean delivery, but it might not be a clinically useful predictor. Moreover, Kuwata et al. demonstrated that PVI, immediately after anesthesia, was a good predictor of hypotension [19]. Frölich demonstrated that a baseline HR over 90 bpm could be useful to predict post-spinal hypotension [20]. Yokose et al. also demonstrated that a HR of < 71 bpm and > 89 bpm are prognostic values that are useful for predicting hypotension, but other parameters, such as PVI, PI, and HRV, are not useful [21]. Hanss et al. found that changes in HRV may reflect sympatholysis during spinal anesthesia, and preoperative HRV could be a predictor of patients at risk of hypotension after spinal anesthesia [22, 23]. Although some of the above parameters could effectively predict hypotension after spinal anesthesia, in most cases, additional medical appliances were required for detection. However, ultrasound is an essential means, just like anesthesia machine to anesthesiologists, and can easily be accessed by most anesthesiologists. Moreover, in recent years, imaging examinations, such as ultrasound and MRI, have been used to identify aortocaval compression by a gravid uterus. Humphries et al. found that the IVC velocities at the level of origin and at the level of the renal veins were significantly reduced, while that of the azygos vein increased significantly on MRI [24, 25]. This observation was made with the parturient in the supine position, compared with those in the left lateral position, in pregnancies between 34 and 38 weeks of gestation without anesthesia. Fields et al. found that on an ultrasound, 76% of pregnant patients had a maximum IVC diameter in the left lateral tilt position at the level of 2 cm distal to the branching of the hepatic vein [26]. Zieleskeiwicz et al. found the changes in velocity-time integral of subaortic flow, as measured by ultrasound with a cardiac probe, when the parturient was changed from a supine position to a position with their legs elevated could predict hypotension after spinal anesthesia [27].

However, the point of the probe in all the above-mentioned studies was above the proximal part of the IVC compression point, and all these parameters reflected the degree of aortocaval compression indirectly. Xu et al. demonstrated that the right and left toe PI values could effectively predict the incidence of post-spinal hypotension during cesarean delivery [2]. Similar to our study, their observed parameters were also located at the distal part of the IVC compression point, which could effectively indicate the degree of IVC compression and predict the incidence of post-spinal hypotension.

There were two highlights of the ultrasound measurements in this study. First, M-mode ultrasound was used to measure the diameters of the RCFV and IVC. Although B-mode ultrasound is more commonly used for the measurement of vessel diameters, M-mode can display the diameter of vessels at different respiratory phases. In this study, all the diameters of IVC and RCFV were measured during end expiration to eliminate the influence of respiration. Second, we focused on the overall impact of IVC compression on the lower extremity venous system, including both the femoral and saphenous veins. Hence, the measurement site of RCFV was 1 cm proximal to the confluence of the great saphenous vein into the common femoral vein rather than the commonly used measurement site (immediately distal to the confluence of the great saphenous vein into the femoral vein).

Finally, the transverse diameter of the RCFV was found to be associated with hypotension after the spinal anesthesia in this study; however, it is important to note that the BMI of parturients and the neonatal body weight were higher in the Hypotension group than in the No-hypotension group. Therefore, it should be noted that these factors may have more importance to contribute to the hypotension, and the transverse diameter of RCFV may only be a parameter to predict a higher occurrence of hypotension before the spinal anesthesia.

A height of 155–170 cm was selected as a criterion of enrollment to eliminate the height bias that exists with post-spinal hypotension; however, this must be noted as a study limitation. Therefore, the RCFV transverse diameter cut-off value for parturients with a height outside this range needs be researched further.

## Conclusions

We demonstrated that the transverse diameter of the RCFV, as measured on ultrasound, was associated with the occurrence of post-spinal hypotension during elective cesarean delivery and a transverse RCFV diameter of > 12.2 mm could predict parturients at major risk of hypotension during cesarean section, following anesthesia. The transverse diameter of the RCFV, measured by ultrasound, may be a useful predictive method in routine anesthesia practice.

## Data Availability

The datasets generated and analyzed during the current study are available from the corresponding author in response to reasonable requests.

## Abbreviations

IVC: Inferior vena cava
RCFV: Right common femoral vein
OR: Odds ratio
CI: Confidence interval
CSE: Combined spinal epidural
ASA: American Society of Anesthesiology
BMI: Body mass index
IV: Intravenous
SAP: Systolic arterial pressure
HR: Heart rate
UA: Umbilical artery
PI: Perfusion index
IQR: Interquartile range
ROC: Receiver operating characteristic
AUC: Area under the curve
MRI: Magnetic resonance imaging
PVI: Pleth variability index
HRV: Heart rate variability
